# Effect of a mental health nursing competency model: Among Indian students

**DOI:** 10.6026/973206300223050

**Published:** 2026-05-31

**Authors:** Baskaran M, Kavitha J, Jeyadeepa R, Rathi R

**Affiliations:** 1 Lincoln University College, Malaysia and Department of Nursing Research, PSG College of Nursing, Coimbatore, Tamil Nadu, India; 2 Department of Child Health Nursing, PSG Institute of Medical Sciences and Research & PSG Allied Health Sciences, Coimbatore, Tamil Nadu, India; 3 Department of Medical Surgical Nursing, PSG College of Nursing, Coimbatore, Tamil Nadu, India

**Keywords:** Clinical competency, mental health nursing, nursing education, competency-based education, clinical practicum

## Abstract

An evaluation of the impact of mental health nursing competency on the academic and clinical competencies of Indian students. Competency-based education is proposed as a method to bridge the theory-practice gap in nursing. Therefore, it is of interest to evaluate a structured Mental Health Nursing Clinical Competency Model's effect on undergraduate students' academic performance, clinical competency and self-perceived confidence. A quasi-experimental design was used with 98 third-years B.Sc. Nursing students in India, who participated in a 16-week intervention comprising theoretical integration and supervised clinical practicum phases. Outcome measures, including a validated competency checklist, theory exams and OSCE scores, showed significant improvements (p < .001) across all domains, with large effect sizes. Thus, data shows that phased model effectively enhances student outcomes and offers a replicable framework for competency-based mental health nursing education.

## Background:

Competency-based medical education (CBME) represents a paradigm shift from time-dependent training to learner-centered outcomes, reshaping curricula for contemporary practice [1]. Integrating multiple theoretical constructs from behavioral frameworks in primary care health promotion has been shown to enhance intervention effectiveness and health outcomes [2]. In nursing education, innovative pedagogical approaches such as flipped classrooms with team-based learning have demonstrated improvements in student performance despite mixed course evaluations [3]. Global standards for initial nursing and midwifery education aim to elevate educational attainment and ensure equitable role placement, thereby simplifying international recruitment [4]. Whole-team training models, such as Cognitive Analytic Therapy (CAT), have been found to enhance team cohesion and therapeutic confidence in community mental health settings, though they may also increase workload [5]. The global burden of mental disorders, quantified across prevalence and disability metrics, underscores the need for robust educational interventions to address conditions such as depressive and anxiety disorders [6]. Integrated medical curricula require corresponding integrated assessments, including clinical reasoning problems and concept maps, to fully develop critical thinking and clinical skills [7]. However, the broader impact of educational programmes is often constrained by systemic barriers such as insufficient mentorship, infrastructural deficits and heavy workloads, indicating that faculty development alone is insufficient without concurrent nursing education reform [8]. Nursing students frequently encounter significant challenges during clinical training, particularly related to patient and task factors, as well as place-related elements such as clinical site suitability and time availability [9]. Additional barriers during clinical competency evaluations include evaluator negativity, resource shortages, time constraints and inadequate feedback, highlighting the need for targeted interventions [10]. Historical delays in implementing competency-based education have been attributed to poorly defined benchmarks and a lack of evaluation strategies, underscoring the importance of strategic planning and appropriate assessment tools [11]. Online training programmes have shown promise in improving clinician confidence and self-efficacy in rural mental health, though attrition remains a challenge [12]. Simulation-based training has been demonstrated to effectively enhance nursing student confidence and competence, providing a conceptual framework for curriculum development [13]. Skills-based preparation, including simulation of key procedures, is essential for ensuring student readiness before clinical experiences abroad [14]. The competitive "publish or perish" culture further shapes the academic landscape in nursing education, driving scholarly productivity [15]. Interprofessional online programmes have similarly improved rural clinician outcomes, yet retention strategies such as study leave are needed to mitigate attrition [16]. Health professions education continues to evolve as a dynamic field, requiring interdisciplinary research and stakeholder collaboration to address disruptive forces such as pandemics and technological change [17]. A systemic approach encompassing curriculum redesign, innovative teaching methods and robust assessment frameworks is essential for successful integration of competency-based education in nursing [18]. Therefore, it is of interest to report the collective evidence supporting the integration of educational innovations and competency-based strategies in nursing and health professions education.

## Methods:

## Study design and setting:

A quasi-experimental pre-test/post-test design with repeated measures was conducted at PSG College of Nursing, a tertiary institution affiliated with a university teaching hospital in Coimbatore, India, from June to November 2023. The study adhered to the Transparent Reporting of Evaluations with Nonrandomized Designs (TREND) statement guidelines [19].

## Participants:

All third-year B.Sc. Nursing students enrolled in the mental health nursing clinical rotation during the study period were eligible. Total enumeration sampling included 102 students; 98 completed all phases (96.1% retention rate). Inclusion criteria were: (a) regular enrollment in the B.Sc. Nursing program, (b) completion of foundational nursing courses and (c) no prior formal mental health nursing clinical experience. Four students were excluded due to incomplete data from extended medical leave.

## Sample size justification:

Based on a previous competency-based education study [20] reporting moderate effect sizes (Cohen's d = 0.65), an a priori power analysis (G*Power 3.1) indicated that 84 participants would achieve 90% power (α = 0.05, two-tailed) to detect similar effects. The recruited sample (N = 98) exceeded this requirement.

## Intervention: mental health nursing clinical competency model:

The 16-week Mental Health Nursing Clinical Competency Model (MHN-CCM) was developed through a literature review [18] and expert validation by five nursing educators with >10 years' experience. The model comprised two sequential phases:

## Phase I: Theoretical integration (Weeks 1-8):

Eight weekly sessions (3 hours each) integrated theoretical content with active learning strategies. Sessions covered: (a) foundations of mental health nursing and therapeutic communication; (b) psychiatric assessment and mental status examination; (c) therapeutic nurse-patient relationships; (d) psychopharmacology and medication management; (e) cognitive-behavioral interventions; (f) crisis intervention and de-escalation; (g) care of patients with mood disorders; and (h) care of patients with psychotic disorders. Each session combined didactic instruction (1 hour), case-based discussions (1 hour) and skills practice through role-play and simulated scenarios (1 hour). Facilitators included mental health nursing faculty and clinical preceptors.

## Phase II: Supervised clinical practicum (Weeks 9-16):

Eight weeks of supervised clinical experience (24 hours weekly) in inpatient psychiatric units, outpatient departments and community mental health settings. Each student was assigned a dedicated clinical preceptor (registered psychiatric nurse with ≥5 years' experience). Structured learning activities included: (a) direct patient care under supervision, (b) daily preceptor feedback sessions, (c) weekly reflective debriefing groups (2 hours) facilitated by faculty, (d) observed structured clinical encounters with feedback and (e) completion of a clinical competency portfolio documenting achievement of predefined competencies.

## Outcome measures:

Three outcome measures were administered at baseline (T0, Week 1), post-Phase I (T1, Week 8) and post-Phase II (T2, Week 16).

## Clinical competency checklist:

A 45-item instrument assessing competency across six domains: (1) therapeutic communication and interpersonal skills (10 items), (2) psychiatric assessment and formulation (8 items), (3) psychopharmacological management (7 items), (4) crisis intervention and safety management (8 items), (5) psychoeducation and family support (6 items) and (6) professional and ethical practice (6 items). Each item was rated on a 4-point scale (1 = unable to perform, 2 = requires guidance, 3 = competent with minimal guidance, 4 = fully competent independent). Total scores ranged from 45-180. The content validity index was 0.92 based on expert review (n = 7). Inter-rater reliability (ICC = 0.89) was established through independent ratings by faculty and preceptors for 20% of assessments.

## Theory examination:

A standardized 100-mark written examination assessing knowledge of mental health nursing content covered during Phase I. Examinations were developed by faculty not involved in the study and scored independently.

## Objective structured clinical examination:

A six-station OSCE assessing integrated clinical skills including communication, assessment, intervention and crisis management. Each station (10 minutes) was scored using standardized checklists (range 0-10 per station; total 0-60). Stations were validated by expert review and pilot-tested with 10 senior students (Cronbach's α = 0.84).

## Self-Reported confidence:

A single-item rating on an 11-point numerical scale (0 = not at all confident, 10 = extremely confident): "How confident do you feel in your ability to perform mental health nursing care independently?"

## Data collection procedure:

At T0, competency was assessed via standardized simulated scenarios. At T1 and T2, assessment was based on direct observation by preceptors and faculty. All assessors were trained in standardized rating procedures and blinded to prior assessment scores. Theory examinations were conducted under standard examination conditions. OSCEs were administered by faculty not involved in the intervention.

## Ethical considerations:

Ethical approval was granted by the Institutional Ethics Committee, PSG Institute of Medical Sciences and Research (Proposal No: 24/107, Ref: PSG/IHEC/2024/Appr/Exp/157). Written informed consent was obtained from all participants. Confidentiality was maintained through anonymized data coding. Participants could withdraw without academic penalty.

## Statistical analysis:

Data were analyzed using IBM SPSS version 25.0. Normality was assessed using Shapiro-Wilk tests. Repeated-measures ANOVA with Greenhouse-Geisser corrections (where sphericity was violated) and post-hoc Bonferroni tests were applied for continuous variables (competency, theory, OSCE scores). The Friedman test with Wilcoxon signed-rank tests (Bonferroni-corrected) was used for non-parametric confidence ratings. Effect sizes were calculated as partial eta squared (η^2^p) for ANOVA and r for non-parametric comparisons. Statistical significance was set at p < 0.05 (two-tailed) [21].

## Results:

All 98 participants completed the study (mean age = 20.6 ± 1.2 years; 85.7% female; 87.8% with no prior mental health exposure). Chi-square tests revealed no significant differences in gender (χ^2^ = 1.112, p = 0.291), age group (χ^2^ = 2.445, p = 0.485), or prior exposure (χ^2^ = 0.887, p = 0.346) across any baseline measures, indicating sample homogeneity. Table 1 revealed that the clinical competency scores increased significantly across time points (F(2,194) = 121.6, p < 0.001, ηp^2^ = 0.556). Pairwise comparisons confirmed significant increases from T0 (63.1 ± 8.9) to T1 (71.4 ± 7.6; mean difference = 8.3, 95% CI: 6.5-10.1, p < 0.001), T1 to T2 (78.2 ± 7.8; mean difference = 6.8, 95% CI: 5.2-8.4, p < 0.001) and T0 to T2 (mean difference = 15.1, 95% CI: 13.0-17.2, p < 0.001), representing a 24% relative improvement from baseline. Domain-specific improvements were significant across all six competency domains (all p < 0.001). Table 2 Theory examination scores improved from 67.8 ± 8.1 at T0 to 74.9 ± 6.8 at T1 and 79.6 ± 6.2 at T2 (F(2,194) = 89.4, p < 0.001, ηp^2^ = 0.480). All pairwise comparisons were significant (T0-T1: mean difference = 7.1, 95% CI: 5.4-8.8, p < 0.001; T1-T2: mean difference = 4.7, 95% CI: 3.2-6.2, p < 0.001; T0-T2: mean difference = 11.8, 95% CI: 9.9-13.7, p < 0.001), representing a 17.4% overall improvement. Table 3 OSCE scores rose from 33.1 ± 5.5 (66.2%) at baseline to 37.3 ± 4.9 (74.6%) at T1 and 41.2 ± 4.5 (82.4%) at T2 (F(2,194) = 105.2, p < 0.001, ηp^2^ = 0.520), a 24.5% overall improvement. All pairwise comparisons were significant (T0-T1: mean difference = 4.2, 95% CI: 3.1-5.3, p < 0.001; T1-T2: mean difference = 3.9, 95% CI: 2.9-4.9, p < 0.001; T0-T2: mean difference = 8.1, 95% CI: 6.8-9.4, p < 0.001). Table 4 stated that the self-reported confidence increased from a median of 5.0 [IQR: 3.0-6.0] at baseline to 6.0 [5.0-7.0] at T1 and 7.0 [6.0-8.0] at T2 (χ^2^(2) = 85.7, p < 0.001). Wilcoxon signed-rank tests with Bonferroni correction (critical p < 0.017) confirmed significant increases from T0 to T1 (Z = -6.42, p < 0.001, r = 0.65), T1 to T2 (Z = -5.89, p < 0.001, r = 0.60) and T0 to T2 (Z = -8.14, p < 0.001, r = 0.82), representing a 40% median improvement.

## Discussion:

This quasi-experimental study provides evidence supporting the effectiveness of a structured, two-phased Mental Health Nursing Clinical Competency Model in enhancing multiple dimensions of student learning. The 96.1% retention rate underscores the model's acceptability and feasibility within a standard academic semester. The large effect sizes observed (ηp^2^ = 0.48 - 0.56) exceed conventional thresholds for large effects [1] and Enhanced preparation of students for difficult conversations with patients and families demonstrated improvements in academic results [22]. Indicating that 48-56% of the variance in student outcomes was attributable to the MHN-CCM intervention. The phased design offers an understanding of how different educational components contribute to competency development. Phase I successfully built declarative knowledge and introduced psychomotor and affective skills in a low-stakes setting, as evidenced by the significant 8.3-point improvement in competency scores from T0 to T1. Training is key to preparing students for difficult conversations with patients and families [23], which posit that complex skills are best learned through the sequential mastery of components before integration. The shift from passive lectures to active learning strategies optimized cognitive load, allowing foundational competency development without the overwhelming demands of authentic clinical environments. Consistent with our hypothesis, substantial gains occurred during Phase II. The significant 6.8-point further increase in competency scores from T1 to T2 demonstrates that clinical immersion added value beyond classroom achievement. This phase facilitated the transition from "knowing how" to "showing how," central to Miller's pyramid of clinical assessment [24]. The structured practicum, with dedicated preceptors and mandated reflective debriefing, provided essential ingredients for transformative learning: authentic experience, guided supervision and reflective synthesis [25]. These findings challenge curricula that treat theoretical instruction and clinical hours as separate entities rather than as intentionally integrated components. These results align with systematic review an intervention to enhance self-regulated learning demonstrated improvements in academic results [20]. Furthermore, the importance of structured, innovative learning experiences-as highlighted in recent research on faculty roles in simulation [12] resonates with the deliberate design of both phases of our model to build student confidence and competence. The 24.5% improvement in OSCE scores indicates enhanced practical, integrated skill performance. OSCEs represent a gold standard for assessing clinical competence [26] and the improvement suggests students effectively organized and executed skills under observation. The reflective debriefing sessions, a core component of Phase II, likely developed higher-order skills by guiding students to analyze actions, consider alternatives and connect experiences to theory [25]. The convergence between objective competency scores and subjective confidence ratings strengthens the validity of the findings. The 40% improvement in median confidence ratings closely mirrored objective performance gains, suggesting that authentic mastery experiences effectively build self-efficacy per social cognitive theory, this intervention can be integrated into the curriculum as a standardized optional course for higher education students [27]. Increased confidence is a critical precursor to clinical engagement, professional identity formation and career choice [19]. The supportive, structured environment of Phase II likely reduced performance anxiety, allowing students to focus on learning rather than survival. The significant improvements in theory examination scores indicate that clinical application reinforced theoretical understanding, moving beyond rote memorization to applied knowledge. This supports the integrative nature of the MHN-CCM, where classroom learning and clinical practice are mutually reinforcing. This study's context within Indian nursing education demonstrates that substantial educational enhancement is achievable without reliance on expensive high-fidelity simulators, addressing a critical gap in resource-constrained settings [20]. The model provides a pragmatic blueprint for nursing colleges aiming to reform mental health curriculum through optimized use of existing resources.

## Limitations:

This study has several limitations. First, the single-center, quasi-experimental design without a concurrent control group limits generalizability and definitive causal attribution, although the phased progression and large effect sizes strongly support hypothesized effects. Second, the study lacks long-term follow-up to assess competency retention and translation into postgraduate practice. Third, the single-item confidence measurement may not capture the multidimensional aspects of self-efficacy. Fourth, faculty and preceptors delivering the intervention also participated in assessment, potentially introducing bias despite training and blinding procedures. Fifth, the study cannot determine which specific components of the multifaceted intervention contributed most to outcomes.

## Conclusion:

The Mental Health Nursing Clinical Competency Model significantly improves clinical competency, academic performance and self-confidence in undergraduate nursing students by effectively bridging the theory-practice gap through structured, supervised clinical experiences. As an evidence-informed and resource-conscious framework, it prepares practice-ready graduates and warrants further validation through multi-center trials and longitudinal studies.

## Figures and Tables

**Table 1:** Clinical competency scores over time (Mean ± SD)

**Time Point**	**Score (Mean ± SD)**	**Pairwise Comparison**	**Mean Difference**	**95% CI**	**p-value**
T0 (Baseline)	63.1 ± 8.9	T0 → T1	8.3	6.5 - 10.1	< 0.001
T1	71.4 ± 7.6	T1 → T2	6.8	5.2 - 8.4	< 0.001
T2	78.2 ± 7.8	T0 → T2	15.1	13.0 - 17.2	< 0.001

**Table 2:** Theory examination scores over time (Mean ± SD)

**Time Point**	**Score(Mean± SD)**	**Pairwise Comparison**	**Mean Difference**	**95% CI**	**p-value**
T0 (Baseline)	67.8 ± 8.1	T0 → T1	7.1	5.4 - 8.8	< 0.001
T1	74.9 ± 6.8	T1 → T2	4.7	3.2 - 6.2	< 0.001
T2	79.6 ± 6.2	T0 → T2	11.8	9.9 - 13.7	< 0.001

**Table 3:** OSCE scores over time (Mean ± SD)

**Time Point**	**Raw Score (Mean± SD)**	**Percentage (%)**	**Pairwise Comparison**	**Mean Difference**	**95% CI**	**p-value**
T0 (Baseline)	33.1 ± 5.5	66.20%	T0 → T1	4.2	3.1 - 5.3	< 0.001
T1	37.3 ± 4.9	74.60%	T1 → T2	3.9	2.9 - 4.9	< 0.001
T2	41.2 ± 4.5	82.40%	T0 → T2	8.1	6.8 - 9.4	< 0.001

**Table 4:** Self-Reported Confidence Scores at Baseline (T0), T1, and T2

**Time Point**	**Median [IQR]**	**Statistical Comparison**	**Z-score**	**p-value (Bonferroni-corrected)**	**Effect Size (r)**	**Median Improvement**
Baseline (T0)	5.0 [3.-6.0]	T0 → T1	-6.42	< 0.001	0.65	-
T1	6.0 [5.0-7.0]	T1 → T2	-5.89	< 0.001	0.6	-
T2	7.0 [6.0-8.0]	T0 → T2	-8.14	< 0.001	0.82	40%
